# Conflict Entropy-Based Optimization of Vehicle Scheduling in Tunnel Traffic Networks

**DOI:** 10.3390/e28070728

**Published:** 2026-06-25

**Authors:** Yalong Xie, Yuming Liu, Xianhui Nie, Jiaao Guo, Chengfeng Huang

**Affiliations:** 1 School of Economics and Management, Beijing Jiaotong University, Beijing 100044, China; 2 Institute of Computing Technology, China Academy of Railway Sciences Corporation Limited, Beijing 100081, China; 3 Beijing Jingwei Information Technology Co., Ltd., China Academy of Railway Sciences Corporation Limited, Beijing 100081, China; 4 School of Civil Engineering, Beijing Jiaotong University, Beijing 100044, China

**Keywords:** tunnel traffic network, conflict entropy, vehicle timetable scheduling, path planning, improved social force model

## Abstract

Against the backdrop of the advancing Transportation Power Strategy, long and large tunnels face critical challenges in ensuring the safety and efficiency of transportation scheduling due to their harsh environment, complex traffic network, and the need for coordination among multiple types of vehicles. Addressing the shortcomings of existing research—such as the disconnection between path planning and dynamic environments, insufficient coordination between timetables and paths, and incomplete conflict management—this paper constructs a comprehensive optimization model for the scheduling of construction vehicles in tunnel traffic networks. Firstly, integrating the improved social force model with the BPR function, an adaptive social force-BPR path planning model with a collision compensation mechanism is proposed, and the weights of sub-items are optimized using the improved AHP algorithm. Secondly, a constraint system covering paths, spatio-temporal logic, and three types of conflicts (crossing conflicts, head-on conflicts, and congestion conflicts) is established, and a bi-objective function of “minimum total scheduling time” and “minimum number of conflicts” is designed. Combined with the improved NSGA-II algorithm, the collaborative optimization of departure intervals and paths is realized. In particular, a conflict entropy repair operator is introduced to quantify the conflict chaos through node conflict entropy and vehicle conflict entropy, and the scheduling strategy is accurately adjusted based on the logic of “priority ranking-dynamic delay” to balance conflict resolution and efficiency loss. Finally, a case verification is carried out relying on a tunnel topological network with 30 nodes and 41 edges. The experimental results show that the optimal repulsion coefficient *k_f_* of the social force model is 20, and the maximum departure interval of 8 min is the best configuration after introducing the repair operator. At this time, the total scheduling time is 136 min, and the total number of conflicts is only 2, completely avoiding high-risk head-on conflicts and congestion conflicts. The research outputs a vehicle scheduling scheme, enriches the theory of tunnel traffic scheduling, and provides scientific and feasible technical support for the coordinated scheduling of construction vehicles in long and large tunnels.

## 1. Introduction

With the continuous advancement of the Transportation Power Strategy, long and large tunnels (length exceeding 1.00 km), as key components of transportation infrastructure, are witnessing increasing construction scale and difficulty. Due to the harsh internal environment, complex road topology, and diverse needs such as muck transportation and personnel transportation during the construction period, the orderly scheduling of various types of construction vehicles has become the core link to ensure construction safety and efficiency [1,2,3]. The essence of transportation organization in long and large tunnels lies in the collaborative optimization of vehicle paths and timetables. A reasonable path can avoid traffic bottlenecks in complex road networks, and a scientific timetable can reduce vehicle intersection conflicts. The dynamic adaptation of the two is crucial to ensuring the efficient progress of transportation tasks [4]. In recent years, academic circles have carried out a series of research on vehicle path planning, timetable collaborative optimization, and optimization algorithm improvement, providing certain theoretical support for tunnel transportation scheduling.

In terms of vehicle path planning, Tian et al. [5] constructed a two-layer road network data model to meet the path planning needs of special ultra-large vehicles in natural disaster emergency rescue, integrating an improved shortest path algorithm with bridge and tunnel weight and height constraints and a parallel bidirectional Dijkstra algorithm; Zhang et al. [6] proposed a hybrid path planning algorithm combining A* algorithm and RRT to address the limitations of existing vehicle path planning algorithms, such as slow processing speed and poor path efficiency; Kvitko et al. [7] proposed a new path planning algorithm based on the chaotic behavior of neuron models and with coverage control parameters. Experimental verification shows that compared with similar chaotic path planning methods, it can complete regional coverage faster and more evenly; Tong et al. [8] proposed a multi-vehicle path planning method based on evolutionary game (EG-MVRP), which forms a collaborative optimization strategy by constructing a vehicle grouping model and analyzing the interest conflicts and coordination needs among groups, effectively alleviating local congestion and improving the overall operational efficiency of the traffic network. However, current research mainly focuses on perspectives such as algorithm fusion optimization [9,10,11] and multi-vehicle collaborative mechanisms (game evolution) [12,13,14,15], providing diversified solutions for vehicle path planning and effectively solving core needs such as efficiency improvement [16,17] and congestion alleviation [18] in different scenarios. Nevertheless, most existing studies focus on ordinary open road networks or general emergency scenarios and are insufficiently adaptable to the special scenario of long and large tunnel construction, which is closed, dynamic, and multi-constrained. Its scheduling not only relies on path optimization but also needs to realize the dynamic coordination between paths and departure intervals.

The dynamic optimization of departure intervals is the collaborative optimization of timetables. In terms of vehicle timetable optimization, Wang et al. [19] solved the vehicle timetable coordination problem by constructing a two-step optimization framework: the first step uses an integer programming model to rearrange the subway timetable, and the second step establishes an emergency vehicle scheduling model based on the cell transmission model to reduce the total vehicle travel time; Patzner et al. [20] proposed an extended model to solve the public transport flow assignment problem adapted to timetables, which not only ensures capacity-feasible passenger flow assignment results but also incorporates dynamic network congestion effects such as vehicle crowding, passengers being unable to board, and boarding delays; Chen et al. [21] proposed a label-setting shortest path algorithm with timetable and label constraints, constructing a multi-modal urban agglomeration traffic network model including six transportation modes, introducing deterministic finite automata to constrain feasible traffic mode sequences, and adding time window constraints to simulate the static timetable effect of scheduled transportation; Bulková et al. [22] realized the coordinated connection between public transport and railways by constructing an integrated timed-transfer timetable, setting a unified system time slot at the main transfer hub, and matching a new public transport network with standardized paths and regular departures, thereby optimizing operational efficiency and reducing costs. In addition, supported by Intelligent Transportation System (ITS) and vehicle-road collaboration technology [23,24], some studies have introduced real-time traffic perception data, vehicle network communication information, and edge computing architecture to design a distributed timetable [25] dynamic adjustment framework, which can quickly respond to sudden traffic events and road congestion changes, improving the anti-interference ability and execution stability of the timetable.

To meet the demand for improving transfer efficiency in hub-type traffic scenarios, some studies focus on the timetable connection of different transportation modes in comprehensive transportation hubs. By establishing a transfer time minimization model and a unified time slot allocation mechanism, the departure sequence of high-speed rail, intercity rail, urban rail transit, and long-distance passenger transport is optimized, shortening the passenger transfer waiting time and improving the continuity and convenience of travel chains [26,27]. However, most existing timetable collaborative optimization studies focus on open road networks, regular passenger transport, or ordinary freight transport scenarios. For the closed and constrained scenario of long and large tunnel construction with complex road network topology and special vehicle operation characteristics, they have not fully adapted to the operational process constraints of construction vehicles, road network capacity limitations, and the need to avoid multiple types of conflicts (such as crossing conflicts and congestion conflicts). There are still research gaps in the deep coupling optimization mechanism between timetables and paths, and the departure interval adjustment strategy in dynamic environments.

Facing complex scenarios of large-scale traffic networks or multi-agent collaboration, traditional centralized timetable optimization models face problems of low solution efficiency and insufficient robustness. Therefore, some scholars have adopted methods such as decomposition and coordination algorithms and distributed reinforcement learning [28,29] to decompose large-scale optimization problems into local sub-problems and achieve global coordination, improving solution efficiency while ensuring the global optimality and local adaptability of timetable optimization [30,31]. Santanam et al. [32] constructed a data-driven method system and built a data processing pipeline relying on Automatic Fare Collection (AFC) data to generate adjusted train timetables adapted to post-event needs; Shi et al. [33] integrated traffic congestion uncertainty to construct a time-varying network, and realized vehicle path optimization by combining a simulated annealing strategy with a genetic algorithm and optimizing uncertainty through an entropy mechanism to improve global search performance; Mardešić et al. [34] sorted out the evolutionary context from the classic Vehicle Routing Problem (VRP) and Dynamic Vehicle Routing Problem (DVRP) to the Stochastic Dynamic Vehicle Routing Problem (SDVRP), and summarized model-based and model-free reactive solution methods; Yu et al. [35] proposed an Efficient Simulated Annealing (ESA) algorithm, which solved VRPO and showed excellent performance in sixty large-scale test instances. In addition, to meet the demand for scheduling optimization under dynamic constraints, some studies combine real-time environmental perception data with algorithm iteration [36,37] to improve the adaptability of the model to complex scenarios. However, these methods are still difficult to fully adapt to the deep coupling needs of paths and departure intervals in the closed and constrained environment of long and large tunnel construction, nor do they fully cover the collaborative management and control of crossing conflicts, head-on conflicts, and congestion conflicts.

Existing research has formed relatively mature theories and methods for vehicle scheduling optimization in ordinary road networks and regular transportation scenarios, but there are still three core deficiencies for the special scenario of long and large tunnel construction: first, the path planning algorithm is disconnected from the dynamic closed environment of the tunnel, failing to effectively integrate real-time factors such as road congestion and obstacle distribution, and lacking dynamic adaptability; second, the collaborative optimization mechanism between timetables and paths is imperfect, and the traditional “static path + fixed departure interval” model is difficult to avoid multiple types of traffic conflicts, and does not fully consider the operational process constraints of construction vehicles; third, the conflict management and control system is incomplete. Most existing studies focus on crossing conflicts and head-on conflicts, ignoring the risk of node congestion conflicts, and lack an effective characterization of the trade-off relationship between “minimum total scheduling time” and “minimum number of conflicts”.

Therefore, based on the principle of “safety first, efficiency balanced”, this paper constructs a comprehensive scheduling optimization system covering model assumptions, path planning, constraint systems, objective functions, and optimization algorithms. The research aims to achieve three goals: first, propose a path planning model adapted to the dynamic tunnel environment to accurately quantify road travel costs; second, establish a deep coupling collaborative optimization mechanism between paths and timetables to realize the dynamic adaptation of departure intervals and travel paths; third, construct a comprehensive management and control system covering crossing, head-on, and congestion conflicts to efficiently balance scheduling efficiency and safety risks.

## 2. Problem Description

As a key project in transportation infrastructure construction, long and large tunnels feature a complex crisscross internal road network. Composed of entrances and exits, operation demand points, intermediate connection nodes, as well as two-way and one-way road segments, the network includes various types of intersection nodes such as cross-shaped and T-shaped ones. Some road segments (e.g., transverse passages) are narrow with limited traffic capacity. Meanwhile, the internal environment of tunnels is harsh, characterized by low visibility, complex geological conditions, and poor visual environments, which pose challenges to the transportation of construction vehicles [38]. The construction and transportation of such tunnels rely on the coordinated operation of multiple types of vehicles, mainly including muck vehicles and personnel transport vehicles. Responsible for muck transportation and construction personnel transfer, respectively, these two types of vehicles have distinct functions and clear transportation requirements, jointly forming the dense traffic flow inside the tunnel.

The operation process of construction vehicles is as follows: during the outbound trip, vehicles enter the tunnel from the entrance, travel along a specific path to the designated demand point, complete scheduled operations such as muck loading/unloading and personnel boarding/alighting, and then enter the return trip phase to exit the tunnel along the planned path. In this process, tunnel transportation organization must meet two core demands: “safety” and “efficiency”. However, the existing scheduling mode has many urgent problems to be solved, such as:

1. The driving environment inside the tunnel is highly dynamic. Factors such as road segment congestion and temporary obstacle distribution change in real time, directly affecting the expected driving speed and direction of vehicles. The path planning algorithms of existing scheduling models are disconnected from the actual driving environment, failing to comprehensively consider constraints such as road segment congestion and vehicle driving direction, which easily leads to path congestion or conflicts.
2. There is a deep coupling relationship between departure intervals and path selection. Departure intervals determine the traffic density of road segments, while path selection determines the intersection nodes and time periods of vehicles. Traditional scheduling schemes mostly adopt the “static paths + fixed departure intervals” mode, lacking the collaborative optimization of the two. For example, problems such as multiple vehicles converging at the same intersection segment in a short time and oncoming vehicles meeting in one-way segments occur frequently, which not only affect transportation efficiency but also pose potential safety hazards.
3. The complexity of traffic conflicts inside tunnels is far greater than that of ordinary roads. Existing studies mainly focus on crossing conflicts and head-on conflicts, but ignore node congestion conflicts, i.e., when the number of vehicles at a node exceeds the maximum capacity limit within a specific time period, resulting in vehicle detention, a sharp drop in traffic efficiency, and even the risk of chain collisions. Therefore, there is an urgent need to establish a comprehensive conflict resolution mechanism.
4. The scheduling of construction vehicles in long and large tunnels inherently involves a trade-off between two objectives: on the one hand, pursuing “minimum total scheduling time” to ensure construction progress and improve transportation efficiency; on the other hand, achieving “minimum number of conflicts” to avoid safety accidents and protect personnel and equipment safety. These two objectives are often mutually restrictive. Shortening the total time may increase traffic density and conflict risks; excessive conflict avoidance may lead to path detours and prolonged total time. Existing models lack effective characterization and solution of this trade-off relationship, resulting in limited engineering practicality of scheduling schemes.

## 3. Model Construction

### 3.1. Model Assumptions

To simplify the model construction and solution process, clarify the research boundary conditions, and based on the feasibility of engineering practice and the rationality of research logic, the following assumptions are proposed:

1. Assumptions on the Topological Structure of the Tunnel Traffic Network

The internal traffic roads of the tunnel are abstracted as a fixed directed network G=(N,E). Let *N* be the set of nodes, including entrances and exits, demand points, intermediate nodes, etc., and *E* be the set of road segments, including two-way and one-way road segments; the intersection nodes only include cross-shaped and T-shaped types. The physical parameters of nodes and road segments remain constant during the scheduling cycle, with no temporary road occupation or road network damage.

2. Assumptions on Vehicle Characteristics

The ideal driving speeds of muck vehicles and personnel transport vehicles are set to the same constant value to avoid overtaking conflicts. To simplify the model, the physical parameters of the two types of vehicles are consistent, and there is no failure or abnormal parking during driving, maintaining a stable operating state.

3. Assumptions on Driving Rules

Vehicles traveling in the same direction strictly follow the “first-come-first-served (FCFS)” principle—vehicles arriving at a road segment first leave first. The driving route of vehicles has no backflow or loops, and vehicles are not allowed to return to the previous node to avoid conflicts; the outbound path points from the tunnel entrance to the demand point, and the return path points from the demand point to the tunnel exit, with fixed and non-overlapping directions. The maximum line of sight of the driver is a fixed value, and the expected driving direction and speed are dynamically adjusted through the collision compensation mechanism to adapt to the complex driving environment inside the tunnel.

4. Assumptions on Operations and Time

The preparation time of vehicles at the departure point and the operation time at the demand point (such as muck loading/unloading, personnel boarding/alighting) are pre-fixed and do not change with the arrival order; the vehicle departure interval must be within the preset minimum and maximum thresholds, and all transportation tasks must be completed within the preset maximum operation time of the road network.

5. Assumptions on Conflict Determination

Crossing conflicts only occur at crossing road segments, and head-on conflicts only occur on one-way road segments. The two are independent of each other, and the same traffic event is not repeatedly counted in multiple types of conflicts.

In addition, there is no extreme environmental interference during the scheduling cycle, and environmental parameters such as tunnel visibility are stable; the transportation demand (muck volume, number of passengers) is fixed with no sudden additional tasks, and vehicles strictly follow the optimized timetable and path.

### 3.2. Tunnel Muck Transport Vehicles Path Planning Algorithm Based on the Social Force-BPR Model

#### 3.2.1. Adaptive Social Force-BPR Model

The “force” of the social force model [39] is converted into the travel cost of road segments:

(1) $$w_{ij}\;(t)=\alpha \cdot BPR_{ij}\;(t)+\beta \cdot F_{repel,ij}\;(t)+\gamma \cdot (v_{max}\;-v_{ij}\;(t))-\varphi f_{i}^{o}(t)$$

where

$BPR_{ij}\;(t)$—The impedance strength of road segment (*i*, *j*), calculated using the BPR function;

$F_{repel,ij}\;(t)$—The repulsive force of road segment (*i*, *j*) at time *t*—the higher the congestion degree, the greater the repulsive force;

$v_{ij}\;(t)$—The average travel speed of road segment (*i*, *j*) at time *t*; $v_{\max}$ is the maximum speed limit of the road segment—the lower the speed limit, the relatively higher the cost;

$f_{i}^{o}(t)$—The subjective driving force of the vehicle, which determines the driver’s expected speed;

α,β,γ,φ—The weights corresponding to different sub-items.

The BPR function is calculated as follows:

(2) $$BPR_{ij\;}(t)=t_{0,ij}\;\cdot [1+\alpha_{bpr}\;\cdot {\left(\frac{x_{ij}\;(t)}{C_{ij}}\;\right)}^{\beta_{bpr}}\;]$$

where

$t_{0,ij}$—The free-flow travel time of road segment (*i*, *j*);

$x_{ij}\;(t),\;C_{ij}$—The actual flow and capacity of road segment (*i*, *j*), determined by the road width and number of lanes;

$\alpha_{bpr},\;\beta_{bpr}$—Fixed parameters of the BPR function.

The repulsive force of road segment (*i*, *j*) is calculated as:

(3) $$F_{repel,ij}\;(t)=N_{ij}\;(t)\cdot \frac{L_{\left(i,j\right)}}{k_{f}}\;$$

where

$N_{ij}\;(t)$—The number of vehicles on road segment (*i*, *j*) at time *t*;

$k_{f}$—Repulsive coefficient.

The subjective driving force of the vehicle determines the expected speed at which the driver intends to travel. If there are no other vehicles interfering along the route, a vehicle with mass $m_{i}$ will move along the desired movement direction $e_{i}^{0}\;(t)$ towards the destination at the ideal movement speed $v_{i}^{0}\;(t)$. The self-driving force $f_{i}^{o}(t)$ of a pedestrian at time *t* can be expressed as

(4) $$f_{i}^{o}(t)=m_{i}\frac{v_{i}^{0}e_{i}^{0}(t)-v_{i}(t)}{\tau_{i}}$$

where

$v_{i}(t)$—The actual moving speed of vehicle *i* at time *t*; for simplicity, this paper takes $v_{i}(t)=v_{ij}(t)$; the moving speed of the vehicle is equal to its average moving speed on the road segment.

$\tau_{i}$—The time required for vehicle *i* to adjust from the actual speed to the expected speed, i.e., the relaxation time.

When using the traditional social force model, it is found that the calculation of the vehicle’s expected travel direction does not consider the need to real-time change the movement direction due to obstacles in the target direction. It also fails to account for the vehicle’s continuous adjustment of the expected speed according to the surrounding environment, instead setting the expected speed as a constant value. For example, the expected speed will be affected when the travel space inside the tunnel is limited by obstacles.

This paper follows the research method of Zhao and introduces the collision compensation mechanism *d*_1_, determining the safe and effective distance d(α) that the driver can maintain in the α direction within the field of vision

(5) $$d{\left(\alpha \right)}^{2}=d_{\max}^{2}+f{\left(\alpha \right)}^{2}-2d_{\max}f(\alpha )\cos(\alpha_{des}-\alpha )+d_{1}$$

where

$d_{\max}$—The maximum line of sight of the driver;

f(α)—The minimum collision distance between the vehicle and other vehicles or obstacles in the travel direction;

$\alpha_{des}$—The angle between the vehicle’s destination direction and the visual field reference line; due to the unified topological network structure adopted in this paper,$\alpha_{des}=0$ for straight roads and $\alpha_{des}=90$ for turns;

α—The current driving direction of the vehicle;

$d_{1}$—The collision compensation term, calculated as follows:

(6) $$d_{1}\left\{\begin{array}{ll} d_{\max}-f(\alpha ), & f(\alpha )<d_{\max}\\ 0, & f(\alpha )\geq d_{\max} \end{array}\right.$$

The direction corresponding to the minimum value of d(α) in each α direction within the driver’s field of vision is the adaptive expected $d_{1}$ direction for the vehicle’s movement $\alpha^{*}$

(7) $$\alpha^{*}=\arg\min\{d_{i}(\alpha )\}$$

By selecting the ideal speed $v_{i}^{0}$ and the minimum value of the adjusted speed, the adaptive expected speed $v_{des}(t)$ of the vehicle at time *t* can be obtained

(8) $$v_{des}(t)=\min\{v_{i}^{0},d_{h}/\tau_{i}\}$$

where

$d_{h}$—The maximum distance that can be freely moved without obstacles in the optimal direction $\alpha^{*}$. Since the subsequent analysis object of this article is the topological network structure, when $\alpha =\alpha^{*}$ is present, d(α) is $d_{h}$, and $d_{h}$ represents the maximum safe distance in this direction.

Therefore, the subjective driving force of the vehicle at this time is

(9) $$f_{i}^{0}(t)=m_{i}\frac{v_{ij}(t)-v_{des}(t)}{\tau }$$

The introduction of the subjective driving force is of essential necessity for the adaptive social force-BPR model tailored to the tunnel traffic network scenario, as it bridges the gap between microscopic driver behavior characteristics and macroscopic road segment travel cost quantification in the closed and constrained tunnel environment.

Unlike open road networks, where driving behavior is relatively unconstrained, the operation of construction vehicles in long and large tunnels is subject to strict safety rules, such as no overtaking, unidirectional driving in specific segments, and limited sight distance, and drivers must dynamically adjust their expected speed and travel direction based on real-time tunnel conditions while adhering to fixed transportation task objectives. The subjective driving force term precisely captures this active and constrained adjustment behavior of drivers, which cannot be reflected by the BPR impedance term and repulsive force term alone—these two terms only quantify the objective travel impedance caused by road flow-capacity mismatch and vehicle congestion, while ignoring the dynamic response of drivers to the actual driving environment.

Without the subjective driving force, the path planning model would only calculate the static travel cost of road segments and fail to adapt to the dynamic changes in the tunnel traffic environment, leading to a serious deviation between the planned path and the actual operable path for construction vehicles. Moreover, the subjective driving force, combined with the collision compensation mechanism we proposed, can realize the real-time correction of the driver’s expected speed and direction according to the obstacle distribution and safe distance within the maximum sight distance, which makes the calculation of road segment travel cost more in line with the actual driving state of tunnel construction vehicles, and further ensures that the path planning results have both theoretical rationality and engineering practicability. This term thus becomes a key link for the model to achieve dynamic adaptability to the tunnel’s closed and complex traffic environment, and an indispensable component for optimizing the accuracy and applicability of the path planning model for tunnel construction vehicle scheduling.

#### 3.2.2. Calculation of Sub-Item Weights

The traditional Analytic Hierarchy Process (AHP) uses the 1–9 scaling method to determine the relative importance of hierarchical structure elements [40,41]. Although it reduces the requirement for operators’ professional knowledge, its linear scaling method cannot objectively reflect the weights of schemes, introducing significant subjective errors. The resulting inconsistency may undermine the index quantitative ranking function of AHP and affect the accuracy of quantification. Therefore, referring to [42], this paper improves the weight calculation of AHP using the optimal transfer matrix, computes the judgment matrix of the optimal transfer matrix, and calculates weights according to the formula. This avoids the interference of subjective factors on evaluation results and eliminates the need for consistency checking.

Since the social force model has only 4 sub-items, only 3 importance levels are adopted in the importance comparison. An example of the constructed judgment matrix is:

(10) $$A=\left[\begin{array}{cccc} a_{11} & a_{12} & \cdots & a_{1j}\\ a_{21} & a_{22} & \cdots & a_{2j}\\ \vdots & \vdots & \vdots & \vdots \\ a_{i1} & a_{i2} & \cdots & a_{ij} \end{array}\right]$$

where if $a_{ij}=1$, it indicates that *i* is more important than *j*; $a_{ij}=0$ indicates that *i* and *j* are equally important; $a_{ij}=-1$ indicates that *j* is more important than *i*. The optimal transfer matrix of matrix *A* is:

(11) $$R=\left[\begin{array}{cccc} r_{11} & r_{12} & \cdots & r_{1j}\\ r_{21} & r_{22} & \cdots & r_{2j}\\ \vdots & \vdots & \vdots & \vdots \\ r_{i1} & r_{i2} & \cdots & r_{ij} \end{array}\right]$$

where

(12) $$r_{ij}=\frac{1}{n}\sum_{k=1}^{n}\left(a_{ik}-a_{jk}\right)$$

Then the judgment matrix of the optimal transfer matrix is:

(13) $$D=\left[\begin{array}{cccc} d_{11} & d_{12} & \cdots & d_{1j}\\ d_{21} & d_{22} & \cdots & d_{2j}\\ \vdots & \vdots & \vdots & \vdots \\ d_{i1} & d_{i2} & \cdots & d_{ij} \end{array}\right]$$

where

(14) $$d_{ij}=\exp(r_{ij})$$

The theoretical weight of a single factor is:

(15) $$W={\left[w_{1},w_{2},w_{3},\cdots ,w_{i}\right]}^{T}$$

(16) $$w_{i}=\frac{\sqrt[{n}]{\prod_{k=1}^{n}d_{ik}}}{\sum_{k=1}^{n}\sqrt[{n}]{\prod_{k=1}^{n}d_{ik}}}$$

This improved AHP framework effectively reduces the subjective deviation of parameter determination and eliminates the systematic error introduced by the traditional method from the two core dimensions. On the one hand, it abandons the 1–9 linear scaling method widely used in traditional AHP, which cannot objectively reflect the relative importance of indicators and is prone to introducing significant subjective errors in the scoring process. The 3-level importance grading system adopted in this study narrows the optional range of importance comparison, reduces the subjective randomness of decision-makers in the pairwise comparison process, and avoids the distortion of importance judgment caused by the excessive span of the scaling system. On the other hand, the optimal transfer matrix-based correction method fundamentally eliminates the need for consistency checks and repeated manual adjustments of the judgment matrix in the traditional AHP workflow. In the traditional method, when the judgment matrix fails the consistency test, researchers need to subjectively adjust the matrix elements repeatedly, which will bring secondary subjective deviation and affect the objectivity of the weight results. The method proposed in this paper directly obtains the corrected judgment matrix with inherent consistency through the optimal transfer matrix transformation, which avoids the subjective interference caused by manual adjustment, and ensures that the final weight parameters are derived from strict mathematical calculation rather than subjective revision, thus systematically eliminating the subjective error in the parameter determination process.

#### 3.2.3. Conflict Repair Operator

Based on the entropy logic of “chaos quantification—priority ranking—dynamic delay”, this section designs an entropy-driven timetable delay conflict repair operator. It quantifies the uncertainty of system conflict distribution through conflict entropy and accurately matches the delay priority and duration, ensuring both the effect of conflict resolution and reducing efficiency loss caused by blind delay. Two entropy values are defined in this section: node conflict entropy and vehicle conflict entropy.

Node conflict entropy measures the conflict chaos of a single node. The conflict entropy of node *i* is the probability distribution entropy of various conflicts (crossing conflicts, head-on conflicts, congestion conflicts):

(17) $$H_{N}(i)=-\sum_{m=1}^{3}p_{i,m}\ln p_{i,m}$$

where

$p_{i,m}$—representing the ratio of the number of Cm-type conflicts at node *i* to the total number of conflicts at node *i*; if the total number of conflicts is 0, the conflict entropy is 0.

To highlight the priority of bottleneck nodes, the node weight is corrected:

(18) $$H_{N}^{w}(i)=w(i)\cdot H_{N}(i)$$

where

w(i)—is the node importance weight, taking 1.2 for bottleneck nodes and 1.0 for ordinary nodes.

Vehicle conflict entropy is used to quantify the chaos of vehicle conflict correlation, mainly reflecting the distribution characteristics of conflicting nodes. A higher entropy value indicates that the vehicle’s conflicts cover a wider range and have more complex correlations.

(19) $$H_{V}(k)=\sum_{i\in S_{k}}q_{k,i}\cdot H_{N}^{w}(i)$$

where

$S_{k}$—the set of all conflicting nodes involved in vehicle *k*;

$q_{k,i}$—the conflict proportion of vehicle *k* at node *i*:

(20) $$q_{k,i}=\frac{M_{k,i}}{M_{k}}$$

where

$M_{k,i}$—the number of conflicts of vehicle *k* at node *i*;

$M_{k}=\sum_{i\in S_{k}}M_{k,i}$—is the total number of conflicts of vehicle *k*.

Based on the above entropy indicators, a “priority ranking—dynamic delay” repair logic is designed to ensure accurate and efficient timetable delay behavior:

Step 1: Calculate the $H_{N}^{w}(i)$ values and conflict vehicle $H_{v}(k)$ values of all conflicting nodes, and sort them in descending order based on $H_{v}(k)$.

Step 2: Define the median of the conflicting vehicle entropy values as the delay priority threshold $H_{th}$. For vehicles with high entropy values, re-plan their paths and select another path according to the improved Logit model; for vehicles with low entropy values, delay the departure interval by a random interval of 1~4 min.

Step 3: After modifying the vehicle’s travel path and departure interval, recalculate the node and vehicle conflict entropy. If the new entropy value $H_{\mathrm{new}}\geq H_{\mathrm{old}}$, indicating an increase in the chaos of the new vehicle scheduling scheme, adjust the delay duration until the new conflict entropy decreases.

The improved conflict repair operator integrated with conflict entropy avoids the “experience-based delay” of the original operator. It uses entropy values to accurately locate vehicles that need priority handling and dynamically adjusts the duration, with the delay duration positively correlated with conflict chaos. This not only ensures conflict resolution but also reduces invalid waiting; through entropy feedback verification, it ensures that the overall system chaos after delay decreases rather than local conflict transfer.

Beyond the optimization of the repair operator itself, the introduction of conflict entropy is of core theoretical and practical importance to the entire scheduling optimization framework constructed in this paper. First, it fundamentally breaks through the limitation of traditional conflict metrics that only rely on frequency counting. Unlike conflict frequency, which can only reflect the total number of conflict events, conflict entropy can effectively characterize the spatial distribution, type heterogeneity, and risk correlation of conflicts, distinguishing the essential difference between high-risk concentrated conflicts at bottleneck nodes and low-risk dispersed conflicts, which is a key supplement to the safety risk characterization system of tunnel traffic networks. Second, conflict entropy establishes a quantifiable trade-off mechanism between the dual optimization objectives of minimum total scheduling time and minimum conflict number. It provides a clear and measurable gradient for algorithm iteration, avoiding the blind trade-off between efficiency and safety in traditional models, and ensuring that the optimized scheduling scheme can balance construction efficiency and operational safety in a more scientific and controllable manner.

Furthermore, for the closed and constrained environment of long and large tunnel construction where conflicts are often highly concentrated at a small number of critical nodes, conflict entropy can accurately locate the core risk sources of the network, effectively preventing the problem of local conflict transfer in conventional scheduling adjustment, and ensuring that conflict resolution measures can truly reduce the overall systemic risk rather than just shifting the spatial position of conflicts.

### 3.3. Constraints and Objective Function

The subsequent descriptions of formulas and variables in the article are shown in Table 1.

#### 3.3.1. Vehicle Travel Path Constraints

When path *l* passes through node interval (*i*, *j*), it must pass through node *i* and node *j* inevitably [43].

(21) $$x_{ij}^{l}\leq y_{i}^{l}\;\;\;\;\;\forall l\in L\;,i,j\in N,\;(i,j)\in E$$

(22) $$x_{ij}^{l}\leq y_{j}^{l}\;\;\;\;\;\forall l\in L\;,i,j\in N,\;(i,j)\in E$$

(23) $$x_{ij}^{l}=x_{ji}^{l}\;\;\;\;\;\forall l\in L\;,i,j\in N,\;(i,j)\in E$$

Each muck vehicle can and can only pass through one predetermined collection of sections with muck transportation demand points, and each personnel transport vehicle can and can only pass through one predetermined collection of sections with passenger demand points.

(24) $$\sum_{\{i,j\}\in A_{m}^{u}}x_{\left\{i,j\right\}}^{k}=1,\;\;\;\;\;\forall k\in K_{m}$$

(25) $$\sum_{\{i,j\}\in A_{p}^{u}}x_{\left\{i,j\right\}}^{k}=1,\;\;\;\;\;\forall k\in K_{p}$$

Vehicles can only move forward through existing roads; that is, when a vehicle’s path passes through node interval (*i*, *j*), the tunnel network it is in must pass through node interval (*i*, *j*), and vice versa.

(26) $$x_{ij}\leq \sum_{l\in L}x_{ij}^{l}\;\;\;\;\;\forall l\in L,(i,j)\in E$$

(27) $$x_{ij}^{l}\leq x_{ij}\;\;\;\;\;\forall l\in L,(i,j)\in E$$

Within the tunnel network, the “inflow = outflow” principle of network flow is followed: vehicles enter from the entrance and exit from the exit, with no traffic flow stagnation at intermediate nodes.

(28) $$\sum_{\{i,j\}\in A}x_{i,j}^{k}-\sum_{\{j,i\}\in A}x_{j,i}^{k}=\left\{\begin{array}{c} -1,\;j=0\\ 1,\;j=n\\ 0\;j\neq 0,n \end{array}\right.\;\;\;\;\forall k\in K$$

The traveling direction of any vehicle on path *l* is unidirectional, and backflow is not allowed in the path. For safety reasons, vehicles will not return to the previous node to avoid other vehicles.

(29) $$x_{\left(i,j\right)}^{l}+x_{\left(j,i\right)}^{l}\leq 1\;\;\;\;\;\forall l\in L,(i,j)\in E$$

In addition, no loops are allowed in the travel route of any vehicle on the path.

(30) $$\sum_{\{i,j\}\in E}x_{ij}^{l}+1=\sum_{i\in N}y_{i}^{l},\;\;\;\;\;\forall l\in L,i\in N$$

(31) $$\sum_{i\in B}\sum_{j\in B}x_{ij}^{l}\leq \left|B\right|-1,\;\;B\in N,\;\left|B\right|\geq 2,\;\;\;\;\;\forall l\in L,(i,j)\in E$$

#### 3.3.2. Vehicle Spatiotemporal Logic Constraints

The vehicle departure intervals must be within the preset upper and lower limits, and the departure time of the *k*-th vehicle is the sum of the departure intervals of the first *k* vehicles.

(32) $$\Delta t_{\min}\leq \Delta t_{dep,k}\leq \Delta t_{\max}\;\;\;\;\;\forall k\in K$$

(33) $$T_{arr,(0,j),k}=\sum_{m=1}^{k}\Delta t_{dep,m}\;\;\;\;\;\forall (0,j)\in E,k\in K$$

The vehicle’s running time is divided into actual road segments and virtual road segments. The actual road segment is the ratio of the physically passed road to the ideal speed; the virtual road segment refers to the “working” time when the vehicle arrives at the destination.

(34) $$t_{run,(i,j),k}=\frac{L_{\left(i,j\right)}}{v_{i}^{0}}\;\;\;\;\;\forall (i,j)\in E,k\in K$$

(35) $$t_{run,i,k}=t_{op,i}\;\;\;\;\;\forall i\in N,k\in K$$

Vehicles strictly follow the first-come, first-leave, and first-come-first-served (FCFS) principles.

(36) $$arr_{(i,j),k}(t)\geq dep_{(i,j),k}(t),\;\;\;\;\;\forall (i,j)\in E,k\in K$$

(37) $$arr_{(i,j),k}(t)\geq arr_{(i,j),k}(t-1),\;\;\;\;\;\forall (i,j)\in E,k\in K$$

(38) $$dep_{(i,j),k}(t)\geq dep_{(i,j),k}(t-1),\;\;\;\;\;\forall (i,j)\in E,k\in K$$

(39) $$T_{arr,(i,j),k}=t\cdot [arr_{(i,j),k}(t)-arr_{(i,j),k}(t-1)],\;\;\;\;\;\forall (i,j)\in A,k\in K$$

(40) $$T_{dep,(i,j),k}=t\cdot [dep_{(i,j),k}(t)-dep_{(i,j),k}(t-1)],\;\;\;\;\;\forall (i,j)\in A,k\in K$$

The transportation demand of vehicles must be satisfied

(41) $$\begin{array}{l} arr_{i,k}(t)\cdot C_{p}\geq D_{i},\;\;\;\;\;i\in N_{p}\\ arr_{i,k}(t)\cdot C_{m}\geq D_{i},\;\;\;\;\;i\in N_{m} \end{array}$$

#### 3.3.3. Vehicle Conflict Constraints

To simplify the model calculation, this paper sets the ideal operating speeds of personnel transport vehicles and muck vehicles to the same value, so that overtaking conflicts will not occur.

① Crossing Conflicts

**Figure 1:**
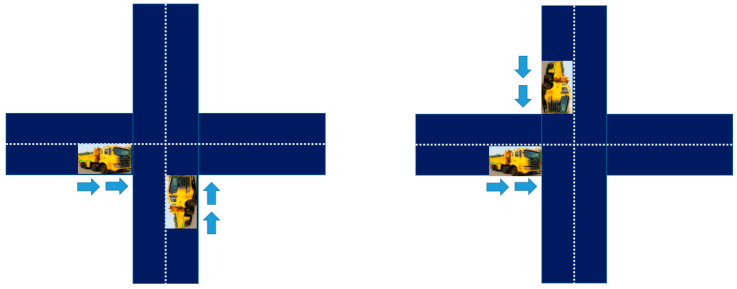
Schematic diagram of tunnel crossing conflicts.

A crossing conflict is considered to occur when two vehicles meet at an intersection, as shown in Figure 1. When the intersection is relatively wide, it can be considered that no crossing conflict occurs, and the actual situation of the tunnel entrance should be analyzed.

(42) $$\begin{array}{l} \left|T_{arr(i,j),k}(t)-T_{arr(i,j^{\prime }),k^{\prime }}(t)\right|+(1-x_{\left(i,j\right)}^{l})\Omega +(1-x_{\left(i,j^{\prime }\right)}^{l^{\prime }})\Omega \geq \Delta t_{c}\\ (i,j)\in E_{c},k,k^{\prime }\in K(k\neq k^{\prime }),l,l^{\prime }\in L \end{array}$$

For safety, the number of crossing conflicts between vehicles in the tunnel must be controlled within a certain range:

(43) $$C_{1}=\sum_{(i,j)\in E_{c}}\sum_{k,k^{\prime }\in K,k\neq k^{\prime }}x_{k,k^{\prime }}^{cross}(i,j)\leq {C_{1}}^{\prime }$$

where

$x_{k,k^{\prime }}^{cross}(i,j)$—0–1 variable, which is 1 when a conflict occurs between vehicle *k* and vehicle *k*’ on the crossing road segment (*i*, *j*).

② Head-on Conflicts

**Figure 2:**
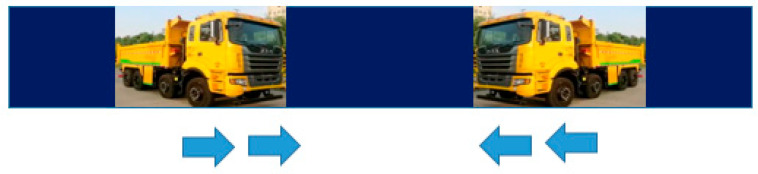
Schematic diagram of tunnel head-on conflicts.

For some narrow transverse passages, only one-way traffic is allowed, so head-on conflicts must not occur. As shown in Figure 2.

For safety, the number of head-on conflicts between vehicles in the tunnel must be controlled within a certain range:

(44) $$C_{2}=\sum_{(i,j)\in E_{s}}\sum_{k,k^{\prime }\in K,k\neq k^{\prime }}x_{k,k^{\prime }}^{\mathrm{oppose}}(i,j)\leq {C_{2}}^{\prime }$$

where

$x_{k,k^{\prime }}^{\mathrm{oppose}}(i,j)$—0–1 variable, which is 1 when a conflict occurs between vehicle *k* and vehicle *k*’ on the one-way road segment (*i*, *j*).

③ Congestion Conflicts

**Figure 3:**
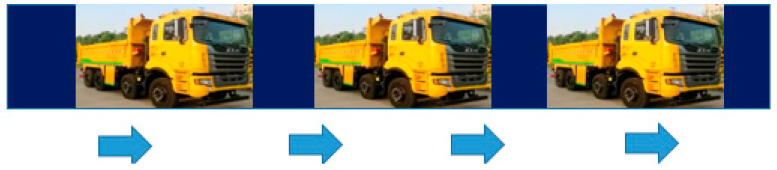
Schematic diagram of tunnel congestion conflicts.

Whether it is a cross-shaped passage, a T-shaped passage or other types of passages, too many vehicles shall not accumulate at the same node at time *t*. As shown in Figure 3.

For safety, the number of congestion conflicts between vehicles in the tunnel must be controlled within a certain range:

(45) $$I(i\in N_{c})\cdot n_{i,t}\leq C_{\max}$$

(46) $$C_{3}=\sum_{i\in N_{c}}\sum_{t}x_{i,t}^{crowd}\leq {C_{3}}^{\prime }$$

where

$x_{k,k^{\prime }}^{crowd}(i,j)$—0–1 variable, which is 1 when the number of vehicles at node *i* exceeds the capacity at time *t*, otherwise 0.

#### 3.3.4. Vehicle Scheduling Objective Functions

This paper adopts a two-layer optimization approach. On the premise of ensuring system safety, the transportation organization scheme for multi-type construction vehicles in the tunnel designs the departure timetable of construction vehicles, reasonably arranges the operation paths and completes various transportation tasks as efficiently as possible. The two objective functions are minimizing the total vehicle scheduling time and minimizing the total number of conflicts.

① Minimizing the Total Vehicle Scheduling Time

(47) $$\min Z_{1}=\sum_{k\in K}\left[\sum_{l\in L}x_{k}^{l}\cdot \sum_{(i,j)\in l}\frac{L(i,j)}{v_{i}^{0}}+\sum_{i\in D}x_{k,i}^{\mathrm{demand}}\cdot t_{op,i}\right]$$

where

*D*—Set of all demand points, $D=N_{m}\cup N_{p}$

② Minimizing the Total Number of Conflicts

This paper defines the total number of conflicts as the sum of three types of conflicts, i.e., total number of conflicts = number of crossing conflicts + number of head-on conflicts + number of congestion conflicts.

(48) $$\min Z_{2}=C_{1}+C_{2}+C_{3}$$

#### 3.3.5. Vehicle Scheduling Optimization Algorithm Based on Improved NSGA-II

To address the dual-objective optimization requirements of “minimum total travel time” and “minimum number of conflicts” in the transportation organization of multiple types of construction vehicles in long and large tunnels, combined with the engineering characteristics of complex road network topology, frequent traffic flow interactions, and strict spatio-temporal constraints in tunnels, a collaborative optimization algorithm based on the Non-dominated Sorting Genetic Algorithm (NSGA-II) is designed. This algorithm fully draws on the path-time coupling optimization idea of the Adaptive Large Neighborhood Search (ALNS) algorithm in previous studies, integrates the core advantages of NSGA-II in balancing the convergence and diversity of solution sets in multi-objective optimization, and realizes the collaborative optimization of dual objectives through coding design, genetic operation adaptation, non-dominated sorting, and elite retention mechanisms, providing scientific decision support for the transportation organization of tunnel construction vehicles.

The detailed steps of the optimization algorithm are as follows:

Step 1: Coding Design

This paper takes the vehicle departure intervals as the initial population and randomly generates p groups of initial populations. Each population represents a departure scheme $\left(\Delta t_{dep,k1},\Delta t_{dep,k2},\Delta t_{dep,k3},\ldots ,\Delta t_{dep,kp}\right)$, and the value of any gene in the departure scheme must satisfy $\Delta t_{\min}\leq \Delta t_{dep,k}\leq \Delta t_{\max}\;$.

Step 2: Decoding process

**Figure 4:**
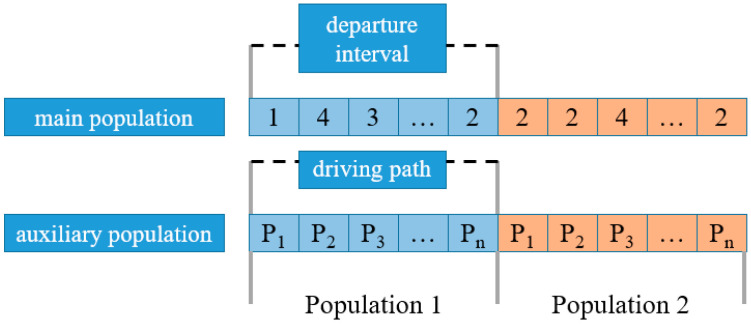
Chromosome encoding schematic diagram.

The core of decoding is based on the gap encoding between transmissions. By improving the social force model, the driving paths (including return paths) of vehicles are dynamically derived, and the values of the dual objective functions are accurately calculated. This ensures that the decoding results are completely consistent with the model and constraints in the paper. The specific operation is as follows:

① Extract the interval $\Delta t_{dep,k}$ from the population encoding, and based on the constraints in the previous text, derive the departure time sequence $Time_{k}$, clearly determining the departure time for each vehicle. ② Path dynamic derivation: Firstly, determine the candidate road segment set. Based on the tunnel traffic topology network, filter all candidate road segments that meet the path constraints to form a unified candidate road segment set. Then, calculate the road passage cost for each candidate road segment (*i*, *j*), based on the improved social force model of Equations (1)–(16), and calculate the passage cost of each path at time *t*. Finally, select the optimal path. With the goal of minimizing the “total co-travel cost”, choose the optimal driving path and return path for each vehicle *k*. ③ Constraint verification: Check whether the derived vehicle routes satisfy all the constraints (time constraints, spatial constraints, logical constraints, etc.) mentioned in the paper. If there are violations of constraints, such as the presence of loops in the route, then the route selection should be adjusted based on the improved social force model until all constraints are met. The vehicle’s driving path will be added as an additional population to the initial population, as shown in Figure 4. Here, $P_{1}=[N_{11},N_{12},N_{10},\ldots ,N_{17}]$ represents the driving path of vehicle 1. ④ Calculate the objective function, and calculate the total driving time of the vehicles and the number of conflicts that occur.

Step 3: Non-dominated sorting and congestion degree calculation

Using the classic sorting mechanism of NSGA-II, the population individuals are evaluated for their superiority and inferiority based on the dual objective function values. For two individuals *X*_1_ and *X*_2_ in the population, if the total travel time of *X*_1_ ≤ that of *X*_2_, and the number of conflicts of *X*_1_ ≤ that of *X*_2_, and at least one objective is strictly better, then *X*_1_ dominates *X*_2_. The distance process of non-dominated sorting is as follows: ① Traverse the population and calculate the dominance count and dominance set for each individual; ② Classify the individuals with a dominance count of 0 into the first non-dominated rank, and this serves as the Pareto optimal frontier; ③ Remove the individuals of the first rank, update the dominance counts of the remaining individuals, classify the new individuals with a dominance count of 0 into the second rank, and so on. Continue this process until the entire population is classified into ranks.

In order to prevent too many individuals within the same hierarchical level from being concentrated, and to ensure the diversity of the solution set, the degree of crowding of each individual in the target space is calculated

(49) $$cd^{i}=\sum_{k}cd_{k}^{i}=\sum_{k}\frac{f_{k}^{i+1}-f_{k}^{i-1}}{f_{k,\max}^{i}-f_{k,\min}^{i}}$$

where

$cd^{i}$—The congestion distance of the *i*-th individual for the *k*-th objective;

$f_{k}^{i+1},\;f_{k}^{i-1}$—Corresponding to the *k*th objective of the (*i* + 1)th individual and the (*i* − 1)th individual, respectively;

$f_{k,\max}^{i},\;f_{k,\min}^{i}$—They represent the maximum and minimum values of the *k*-th objective for the *i*-th individual.

If individuals *i* and *j* have the same ranking, denoted as $p_{rank}^{i}=p_{rank}^{j}$, then the distances $cd^{i}$ and $cd^{j}$ corresponding to these two individuals need to be compared. If condition $p_{rank}^{i}\leq p_{rank}^{j}$ is met and $cd^{i}>cd^{j}$ is true, then the *i*th individual is superior to the *j*th individual.

Step 4: Genetic Manipulation

Taking into account the characteristics of a single code for the inter-activity interval, design cross and mutation operations that are compatible with the constraints, ensuring that the individuals after genetic operations still meet the constraints stipulated in the paper, and at the same time enhancing the evolutionary ability of the population.

① Cross-operation

The cross-operation is divided into two parts. One part is the cross-operation between train intervals, and the other part is the cross-operation between driving paths. The cross-operation of train intervals is as follows:

Step 4-1: Set a type A cross probability $\xi_{1}$ and a type B cross probability $\xi_{2}$, generate a temporary random number ξ. When $\xi \leq \xi_{1}$ occurs, proceed to Step 4-2; when $\xi_{1}\leq \xi \leq \xi_{2}$ occurs, turn to Step 4-3; otherwise, proceed to Step 4-4. In this paper, $\xi_{1}$= 0.4 and $\xi_{2}$ = 0.8.

**Figure 5:**
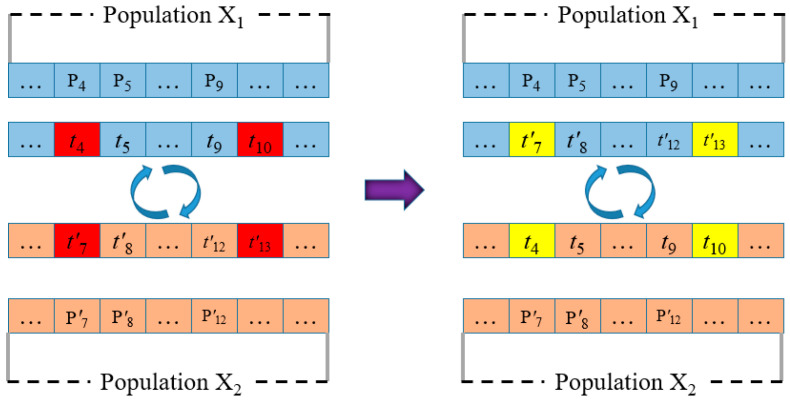
A type of cross-reference illustration 1.

**Figure 6:**
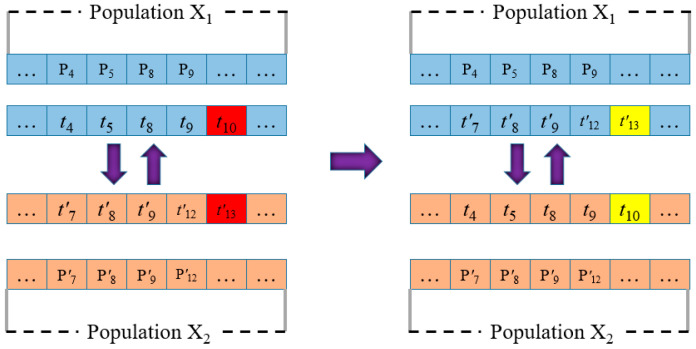
A type of cross-reference illustration 2.

Step 4-2: One type of crossover is the generation of offspring intervals through the crossover of intervals between different parent individuals. A new population is formed by generating intervals between these different parent individuals. Suppose the intervals between these different parent individuals are respectively composed of sets $PL_{1}$ and $PL_{2}$. To ensure the excellence of the crossover result, two vehicle intervals that have conflicts, namely point $pl_{1}\in PL_{1}$ and point $pl_{2}\in PL_{2}$, are randomly selected. Subsequently, these two nodes and all the alleles between them are exchanged and recombined to form a new offspring chromosome, as shown in Figure 5, or the alleles at the front of the conflicting nodes are interchanged, as shown in Figure 6.

**Figure 7:**
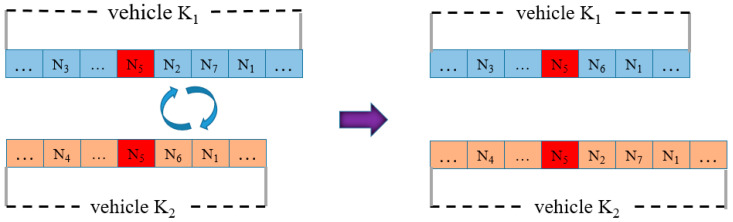
Single-line intersection of vehicle routes.

**Figure 8:**
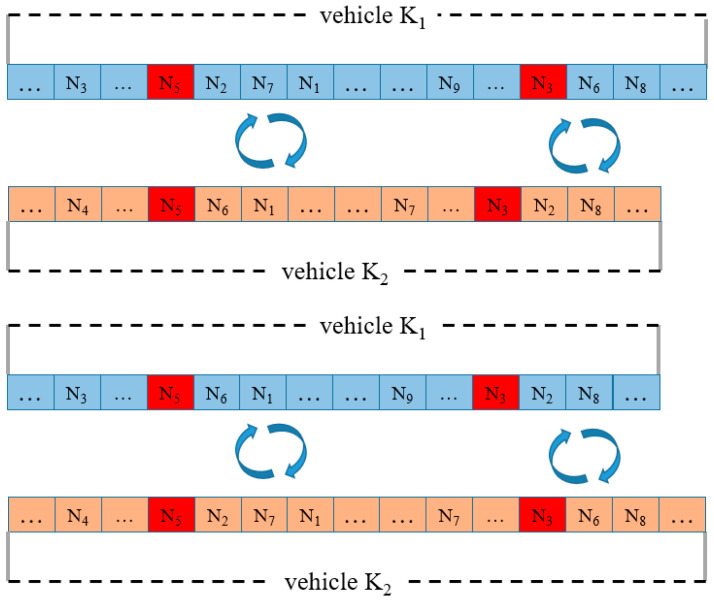
Multiple-line intersection of vehicle routes.

**Figure 9:**
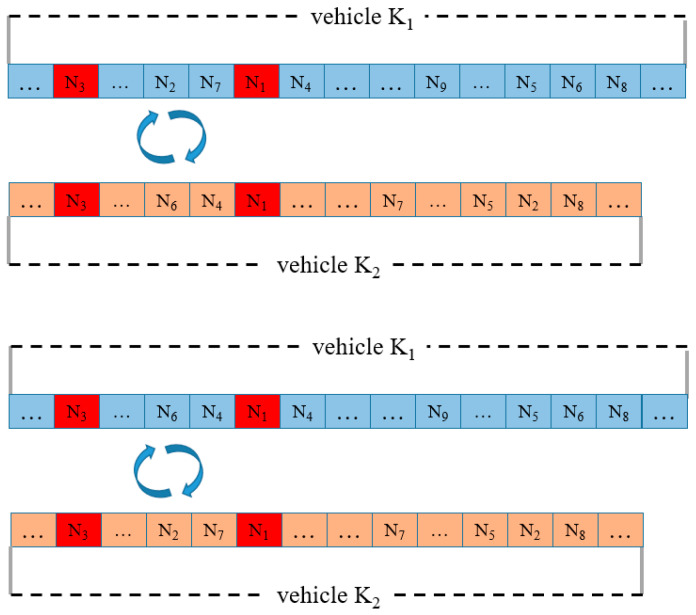
Interchange of intermediate routes in vehicle routing.

Step 4-3: The second type of crossover is the intersection of vehicle travel paths, which does not involve the interval between departures. By using the travel paths corresponding to the same numbered vehicle among different parent generations to generate a new population. Suppose the travel paths of different parent individuals to the same vehicle form sets $PL_{3}$ and $PL_{4}$. Select the conflicting travel nodes $pl_{3}\in PL_{3}$ or $pl_{4}\in PL_{4}$ (different from the departure interval, vehicle travel paths strictly follow the constraints and topological network structure, and it is not necessary for two nodes to have a conflict simultaneously to exchange), and based on the three types of crossover algorithms (single-line crossover, multi-line crossover, and intermediate path interchange), exchange the travel paths of vehicles and generate new offspring chromosomes, as shown in Figure 7, Figure 8 and Figure 9.

The newly formed sub-population obtained through crossover must meet the constraints, such as prohibiting the occurrence of loops or closed circuits in the path. If any violation of the constraints is detected, this crossover will be canceled.

Step 4-4: Proceed to Step 5.

Step 5: Mutation Operation.

**Figure 10:**
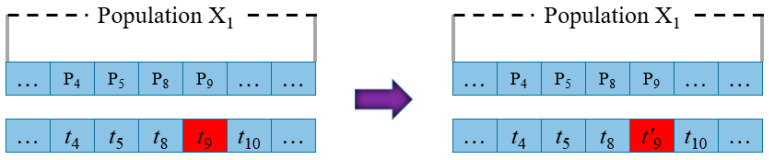
Vehicle departure interval mutation operation.

To ensure the excellence of the mutation results and reduce the complexity of the model, this paper only performs mutation operations on the conflicting departure interval points, extending the departure interval backward by 1 to 4 min (not exceeding the constraint condition of the maximum departure interval), as shown in Figure 10.

Step 6: Elite preservation and population renewal

By merging the parental population and the offspring population, the optimized individuals are obtained, forming a merged population with a size of 2*p*. The non-dominated sorting is conducted for the merged population, and the crowding degree is calculated. Individuals are selected from high to low based on the non-dominated ranking, and the crowding degree within the same ranking is selected from large to small. This process continues until a new population of size *p* is selected, ensuring that high-quality genes are retained and maintaining the diversity of the population. During the iterative process, a part of the population generated after each crossover and mutation is saved as a process solution and incorporated into the calculation of the Pareto frontier solution.

Step 7: Convergence judgment and termination condition

Establish a dual convergence criterion to balance the algorithm efficiency and the quality of the solution: ① Based on the number of vehicles *K*, determine the maximum number of iterations. When *K* ≤ 50, *T* = 150~200 generations; when *K* > 50, T = 200~300 generations. The choice is made according to the actual situation. ② The cross-probability and mutation probability of this paper are 0.9 and 0.1, respectively. ③ When the continuous iteration reaches *N_d_* (*N_d_* = 20~30), the mean change value of the dual objective function for the first non-dominated rank individuals is less than the threshold. In this case, it is considered that the population has converged and the iteration is terminated.

## 4. Case Study

### 4.1. Case Basic Information

**Figure 11:**
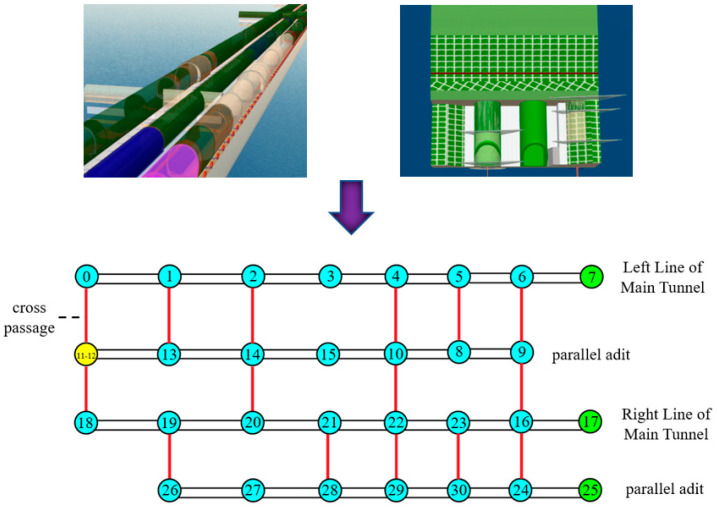
Longitudinal tunnel topological traffic network diagram.

To ensure the convenience and stability of construction links such as tunnel muck discharge and personnel transportation, the construction of long and large tunnel projects has given rise to a diversified road traffic network structure. This has, in turn, promoted the relevant research and technological development on vehicle scheduling and path planning issues within tunnel traffic networks, placing higher requirements on the comprehensiveness and wide adaptability of solutions. To conduct an in-depth exploration of such issues without losing generality, this paper constructs a tunnel traffic network system with a considerable scale and complex topological structure, whose topological form is shown in Figure 11.

This paper assumes that vehicles undergo vehicle inspections at the entrance, so the node at the entrance is double-numbered (11, 12). If regarded as a single node, the network consists of 30 nodes and 41 edges, with road segment lengths set to two specifications: 500 m and 250 m. Among them, channels with a length of 500 m are of the two-way traffic type, while those of 250 m are of the one-way traffic type. To simulate the complex traffic conditions in tunnel construction scenarios, this paper intentionally makes the network topology deviate from a regular grid layout (different from conventional grid-like road networks), so as to further verify and improve the universality and robustness of the proposed method. Based on engineering experience, in the simulation, the traveling speed of muck vehicles and personnel transport vehicles is set to 30 km/h, the minimum vehicle departure interval is 1 min, and the maximum traffic capacities of one-way and two-way road segments are 4 vehicles and 8 vehicles, respectively. That is, at the same time, the maximum capacities corresponding to the congestion constraints of one-way and two-way road segments are 4 and 8.

### 4.2. Calculation of the Optimal Repulsive Coefficient

To determine the optimal repulsive coefficient in path planning, the maximum vehicle departure interval is set to 8 min and the minimum vehicle departure interval to 4 min, with the repulsive coefficient *k_f_* adjusted to 5, 10, 15, and 20, respectively. To avoid the impact of the conflict repair operator on the sensitivity analysis of the *k_f_* coefficient, the repair operator is not considered in the experiment, and only the coding, genetic and mutation operations defined in this paper are adopted. The Pareto optimal frontier solutions under different repulsive coefficients are shown in the following tables, where *nm*_1_ is the total vehicle scheduling time (min), *nm*_2_ is the number of head-on conflicts, *nm*_3_ is the number of crossing conflicts, *nm*_4_ is the number of congestion conflicts, and *nm*_4_ is the total number of conflicts. As shown in Table 2, Table 3, Table 4 and Table 5.

**Figure 12:**
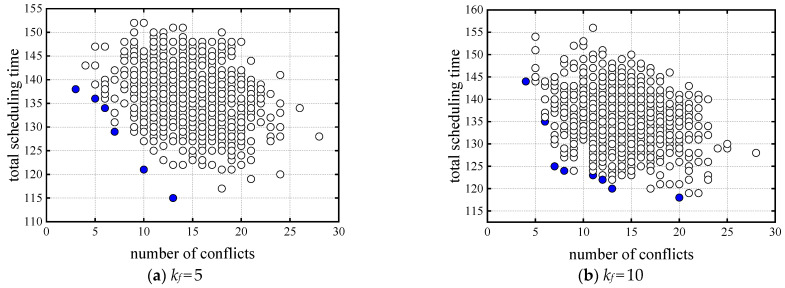

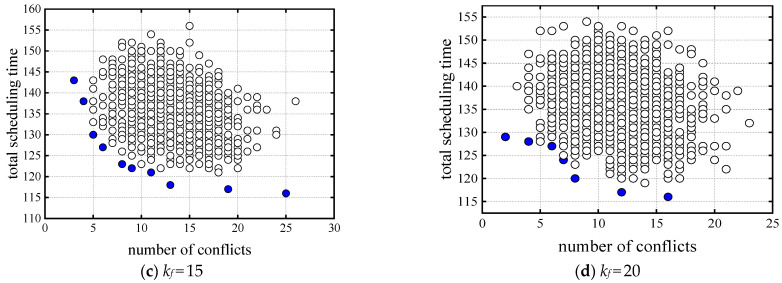
Solutions during the iteration process.

The population solutions generated during the iteration process are shown in Figure 12.

From the calculation results, the Pareto solution set when *k_f_* = 20 is relatively concentrated. The solution numbered 1 achieves a total of only 2 conflicts, which is the minimum value among all repulsive coefficients, with a corresponding total scheduling time of 129 min, ranking among the top in terms of time among the optimal conflict solutions for each *k_f_* value, without significant prolongation of time due to the pursuit of low conflicts. At the same time, as the total scheduling time shortens to 116 min, the total number of conflicts only increases to 16, with a growth rate lower than that of *k_f_* = 10 (20 conflicts corresponding to 118 min) and *k_f_* = 15 (25 conflicts corresponding to 116 min), avoiding the problem of a sharp surge in the number of conflicts.

Compared with other *k_f_* values, although *k_f_* = 5 has a good solution of 3 conflicts corresponding to 138 min, the shortest time of 115 min corresponds to 13 conflicts, showing a steep growth slope of conflicts as time shortens and insufficient flexibility in vehicle scheduling. The total times corresponding to the optimal conflict solutions of *k_f_* = 10 and 15 are 144 min and 143 min, respectively, with 4 and 3 conflicts corresponding thereto, both longer than 129 min of *k_f_* = 20. Moreover, the number of conflicts in the short-time interval is significantly higher, and the proportion of extreme disadvantages in the solution set is higher. Combined with the iterative distribution in Figure 12, the solution set of *k_f_* = 20 is more concentrated in the comprehensively optimal region of total scheduling time and number of conflicts, and the Pareto frontier is closer to the ideal trade-off curve of multi-objective optimization, with a better balance between convergence and diversity. Therefore, this paper concludes that *k_f_* = 20 is the optimal repulsive coefficient balancing total scheduling time and number of conflicts, and *k_f_* = 20 is adopted in the subsequent calculation of vehicle scheduling results.

### 4.3. Vehicle Scheduling Result Calculation

**Figure 13:**
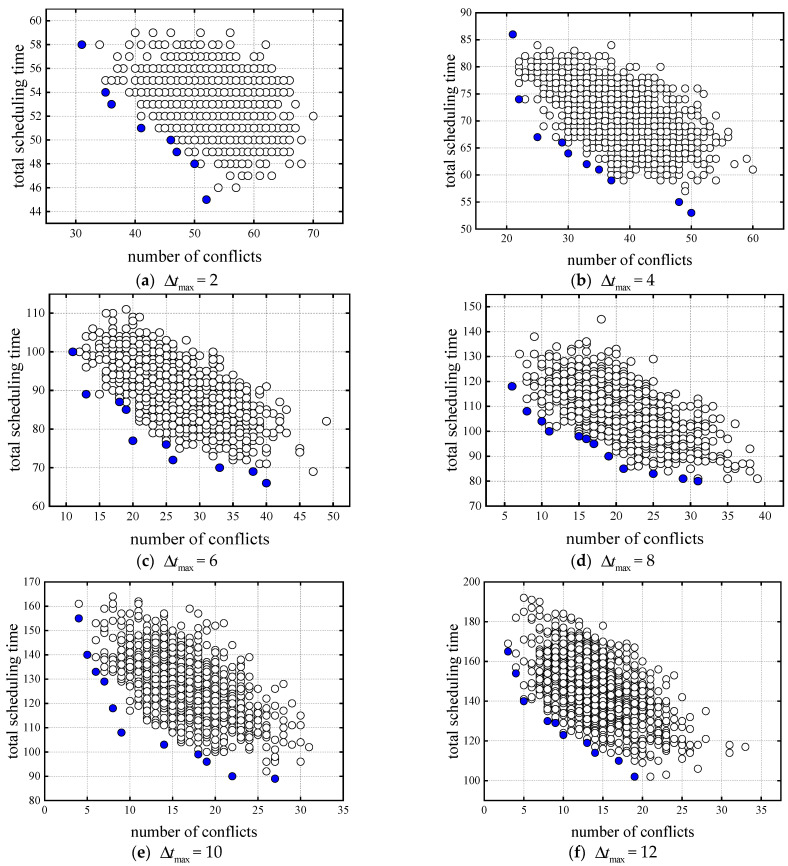
The solutions during different $\Delta t_{\max}$ iteration processes.

To determine the optimal vehicle departure interval, set the minimum departure interval of the vehicles to 1 min, and adjust the maximum departure intervals of the vehicles to $\Delta t_{\max}$ = 2, 4, 6, 8, 10, and 12, respectively. The vehicle scheduling timetable is determined through genetic iteration. To verify the impact of the repair operator on the adaptability of the results, this paper conducts a control experiment. When the repair operator is not considered, the simulation results are shown in Figure 13, and the representative Pareto solutions selected are presented in Table 6.

Taking into account the dual-objective trade-off relationship between the total dispatching time and the total number of conflicts, four optimal maximum intervals of 6 min, 8 min, 10 min, and 12 min were selected. From the data performance, when the maximum interval is 6 min, the total number of conflicts is controlled within 11 to 13 times, and the total time is 89 to 100 min. This ensures transportation efficiency while effectively controlling the conflict risk. At 8 min, the total number of conflicts further decreases to 6 to 10 times, and the total time is 104 to 118 min. The conflict suppression effect is more prominent, and the time increase is within a reasonable range. At 10 min and 12 min, the total number of conflicts is respectively low, at 4 to 7 times and 3 to 5 times. Although the total time is extended, the safety redundancy is significantly improved, which can adapt to scenarios with higher requirements for construction safety. The Pareto solutions corresponding to these four intervals are more concentrated in the target space, and the balance effect of the dual-objective optimization is better. They can cover the tunnel construction scheduling needs with different safety efficiency preferences.

**Figure 14:**
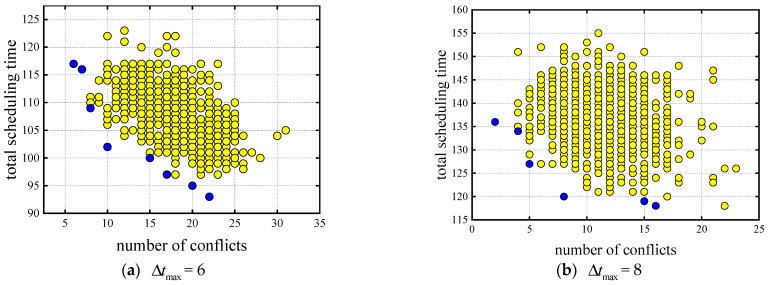

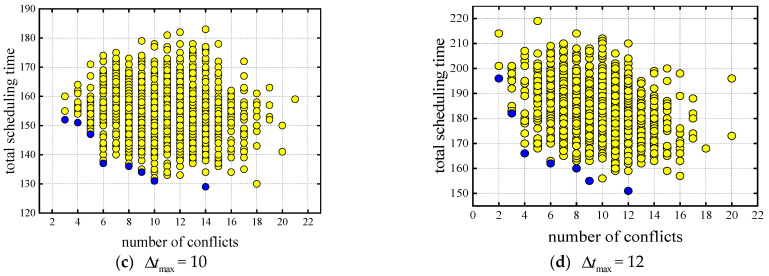
The solutions obtained in different $\Delta t_{\max}$ iteration processes after the introduction of the repair operator.

Subsequently, this paper introduces the repair operator, further refining the collaborative adaptation logic between the transmission interval and the path. The corresponding calculation results after the second simulation are shown in Figure 14, and the representative Pareto solutions selected are presented in Table 7.

In the bi-objective optimization of construction vehicle scheduling for long and large tunnels, after introducing the repair operator, the solution No. 1 with a maximum departure interval of 8 min has basically achieved the dynamic balance between “minimum total scheduling time” and “minimum total number of conflicts”. From the safety dimension, the total number of conflicts of this solution is only 2, which is one of the echelons with the lowest conflict level among all representative Pareto solutions. Moreover, the conflict types are only concentrated on crossing conflicts, with the number of head-on conflicts and congestion conflicts both being 0. This means that head-on conflicts and node congestion conflicts, which are most likely to cause serious accidents inside the tunnel, are completely avoided. Only a small number of crossing conflicts require simple control, resulting in strong safety operability and sufficient safety redundancy in practical engineering.

Compared with the solutions corresponding to other optimal maximum departure intervals, this solution shows an absolute advantage in the trade-off between efficiency and safety: although the solution with a maximum departure interval of 6 min has a slightly shorter total scheduling time, the total number of conflicts reaches 6~7, leading to a significant increase in safety risks; the number of conflicts in the solution set corresponding to 10 min is 3~5, but the total scheduling time is extended to 147~152 min, resulting in obvious efficiency loss; although the total number of conflicts in the solution set of 12 min is also 2~4, the total scheduling time is as long as 166~196 min, excessively sacrificing the construction progress. However, the total scheduling time of the solution selected in this paper is 136 min, which is in a reasonable range. It can not only ensure the efficient progress of construction and transportation tasks but also avoid a sharp surge in conflict risks due to the pursuit of efficiency, perfectly aligning with the engineering principle of “safety first, efficiency balanced” for tunnel construction.

At the same time, the introduction of the repair operator further refines the collaborative adaptation between departure intervals and paths, making the conflict distribution of the solution more concentrated and manageable. The total scheduling time does not have an unreasonable extension due to the improvement of safety redundancy, making it the optimal solution balancing engineering practicality, safety and efficiency.

### 4.4. Vehicle Scheduling Organization Scheme

Based on the optimal parameter combination determined by the above experiments, including the optimal repulsion coefficient kf = 20, maximum departure interval of 8 min, and the introduction of the conflict entropy repair operator, the corresponding Pareto optimal solutions under different production intervals were determined; the vehicle path planning and departure timetable corresponding to the optimal Pareto solution are detailed in Table 8.

### 4.5. Case Conclusions

This section takes a complex, long, and large tunnel topological network with 30 nodes and 41 edges as the simulation scenario, focusing on the dual-objective optimization core of “minimum total scheduling time” and “minimum number of conflicts”. Through experiments such as repulsion coefficient calibration, departure interval optimization, repair operator verification, and scheduling scheme implementation, the effectiveness of the model and algorithm constructed earlier is systematically verified, forming the following core conclusions:

① First, the optimal parameter configuration for path planning is clarified. By comparing the simulation results of four repulsion coefficients (kf = 5, 10, 15, and 20), it is determined that kf = 20 is the optimal repulsion coefficient, under which the Pareto solution set achieves the best balance between convergence and diversity.
② Second, the optimal departure intervals adapted to different engineering scenarios are screened out. Through a control experiment with departure intervals adjusted from 2 to 12 min, it is found that 6 min, 8 min, 10 min, and 12 min are the optimal intervals for dual-objective optimization. The 6 min interval balances transportation efficiency and basic safety; the 10~12 min interval focuses on improving safety redundancy with a relatively small number of conflicts (only 3~7 times); after introducing the repair operator, the 8 min interval achieves the optimal trade-off with a total scheduling time of 136 min and only 2 total conflicts. It completely avoids high-risk head-on conflicts and congestion conflicts, leaving only a small number of easily controllable crossing conflicts, which accurately meets the core requirement of “safety first, efficiency balanced” in tunnel construction and verifies the core value of the repair operator.
③ Third, a vehicle scheduling scheme is output. Based on the optimal parameter combination (kf = 20, maximum departure interval of 8 min, and introduction of the repair operator), the specific departure times, outbound and return paths of 20 construction vehicles are clarified. All paths meet the constraints of no backflow, no loops, and consistency with the road network topology. The timetable strictly follows the “first-come-first-served” principle, which not only ensures the efficient completion of transportation tasks within 136 min but also builds a solid safety barrier through precise conflict management and control, providing a directly referable engineering scheme for the coordinated scheduling of multiple types of construction vehicles in long and large tunnels.

## 5. Conclusions and Future Work

### 5.1. Conclusions

Centering on the core demands of safety and efficiency in the scheduling of construction vehicles for long and large tunnels under the Transportation Power Strategy, this paper addresses key issues such as complex road network topology, dynamic environmental changes, diverse conflict types, and bi-objective trade-offs within tunnels, and constructs a comprehensive scheduling optimization system. By clarifying five categories of model assumptions, including tunnel topology, vehicle characteristics, and driving rules, the research boundaries and applicable conditions are defined; innovatively integrating the dynamic environment adaptability of the social force model with the travel cost quantification capability of the BPR function, an adaptive social force-BPR path planning model is proposed.

A collision compensation mechanism is introduced to realize real-time adjustment of the vehicle’s expected speed and direction, and the improved AHP algorithm is adopted to optimize sub-item weights, effectively reducing the subjective errors of traditional methods; meanwhile, a comprehensive management and control system covering path constraints, spatiotemporal logic constraints, and three types of conflict constraints (crossing, head-on, and congestion conflicts) is established. A bi-objective function of “minimum total scheduling time” and “minimum number of conflicts” is designed, and combined with the improved NSGA-II algorithm, the collaborative optimization of departure intervals and paths is realized. Through constraint-adaptive genetic operations, an elite retention mechanism, and a repair operator, the convergence and diversity of the solution set are guaranteed, and the engineering practicality of the scheme is improved.

Based on the case verification of a complex tunnel topological network with 30 nodes and 41 edges, this paper clarifies the optimal configuration of key parameters: the optimal *k_f_* for path planning is 20, under which the Pareto solution set achieves the best balance between convergence and diversity; the optimal maximum departure interval is 8 min (with the repair operator introduced). Under this configuration, the optimal trade-off of a total scheduling time of 136 min and a total of 2 conflicts is achieved. It not only completely avoids high-risk head-on conflicts and congestion conflicts, but also has a small number of easily controllable crossing conflicts, which accurately conforms to the core principle of “safety first, efficiency balanced” in tunnel construction. Additionally, by outputting the specific departure times, outbound and return paths of 20 construction vehicles, a directly implementable scheduling scheme is formed. All paths meet the constraints of no backflow and no loops, and the timetable strictly follows the “first-come-first-served” principle, effectively solving the prominent problems in traditional scheduling modes such as the disconnection between paths and the environment, the lack of coordination between departure intervals and paths, and incomplete conflict management and control.

The research results of this paper enrich the theoretical system in the field of tunnel traffic scheduling. Engineering-wise, it provides a scientific and feasible technical scheme for the coordinated scheduling of multiple types of construction vehicles in long and large tunnels, which can significantly reduce traffic conflict risks, improve transportation efficiency, and provide support for the high-quality advancement of transportation infrastructure construction.

### 5.2. Future Work

In subsequent work, we will fully incorporate the head-on conflict risks caused by overtaking, yielding maneuvers and temporary vehicle parking on double-lane segments in actual tunnel construction into our research framework. We will supplement a targeted constraint system for such conflicts, expand the calculation dimension of node and vehicle conflict entropy to cover the conflict characteristics of double-lane segments.

Additionally, we will further relax the simplifying assumptions adopted in the current model to break through the limitations of its applicable boundaries and enhance the matching degree between the theoretical model and actual tunnel construction scenarios. For the assumption of identical ideal speeds for muck vehicles and personnel transport vehicles, we will introduce differentiated speed parameter systems in subsequent research, fully considering the speed difference between loaded and unloaded muck vehicles, as well as the priority speed setting of personnel transport vehicles to meet the demand of efficient and timely personnel transfer. We will supplement a complete constraint system for the following conflicts and overtaking conflicts induced by speed heterogeneity, expand the calculation framework of node conflict entropy and vehicle conflict entropy to capture the spatiotemporal distribution characteristics of conflicts caused by speed differences, and optimize the conflict repair operator to achieve accurate identification and efficient resolution of such new conflict types.

## Figures and Tables

**Table 1:** Set of formulas and variables.

Symbol	Definition	Symbol	Definition
(*i*, *j*)	Node interval	{*i*, *j*}	Node section
$x_{\left(i,j\right)}^{l}$	0–1 variable	$y_{i}^{l}$	0–1 variable
*E*	Interval Union Set	*L*	The complete set of all paths
*N*	Set of nodes	*K_m_*	Muck vehicles assembly
$A_{m}^{u}$	Collection of sections with muck transportation demand points	$x_{\left\{i,j\right\}}^{k}$	0–1 variable
*K_p_*	Collection of Transport Vehicles	$A_{p}^{u}$	Collection of sections with passenger demand points
$x_{ij}$	0–1 variable	*K*	All vehicles collection
*B*	The set of any nodes	$\Delta t_{\min}$	Minimum vehicle departure interval
$\Delta t_{\max}$	Maximum vehicle departure interval	$v_{i}^{0}$	Ideal speed of the vehicle
*L*_(*i,j*)_	Length of node interval (*i*, *j*)	$t_{op,i}$	The operation time of requirement point *i*
$arr_{(i,j),k}(t)$	0–1 variable, indicates that vehicle *k* arrives in the interval (*i*, *j*) at time *t*.	$dep_{(i,j),k}(t)$	0–1 variable, indicates that vehicle *k* leaves the interval (*i*, *j*) at time *t*.
$T_{arr(i,j),k}(t)$	The specific time when vehicle *k* arrives at the interval (*i*, *j*)	$T_{dep(i,j),k}(t)$	The specific time when vehicle *k* leaves the interval (*i*, *j*)
$arr_{i,k}(t)$	The number of vehicles arriving at node *i* at time *t*	*D_i_*	The workload of node *i*
*N_p_*	Destination assembly for personnel transportation	*N_m_*	Collection of muck vehicle Destinations
*C_p_*	Passenger vehicle capacity	*C_m_*	Muck vehicle Capacity
*E_c_*	Collection of Intersection Roads	*E_s_*	One-way Road Collection
*N_c_*	The need to limit the set of crowded nodes	*C_max_*	Maximum capacity at the node
$x_{k}^{l}$	0–1 variable, When vehicle *k* selects path *l*, the value is 1.	$x_{k,i}^{\mathrm{demand}}$	0–1 variable, Vehicle *k* is 1 at demand point *i*.

**Table 2:** Pareto optimal frontier solutions generated when *k_f_* = 5.

No.	*nm*_1_	*nm*_2_	*nm*_3_	*nm*_4_	*nm*_5_
1	138	0	3	0	3
2	136	3	2	0	5
3	134	0	6	0	6
4	134	1	5	0	6
5	129	2	5	0	7
6	121	2	8	0	10
7	115	0	13	0	13

**Table 3:** Pareto optimal frontier solutions generated when *k_f_* = 10.

No.	*nm*_1_	*nm*_2_	*nm*_3_	*nm*_4_	*nm*_5_
1	144	0	4	0	4
2	135	2	4	0	6
3	125	0	7	0	7
4	124	1	7	0	8
5	123	2	9	0	11
6	122	0	12	0	12
7	120	0	13	0	13
8	118	1	19	0	20

**Table 4:** Pareto optimal frontier solutions generated when *k_f_* = 15.

No.	*nm*_1_	*nm*_2_	*nm*_3_	*nm*_4_	*nm*_5_
1	143	1	2	0	3
2	138	0	4	0	4
3	130	0	5	0	5
4	127	2	4	0	6
5	123	1	7	0	8
6	122	1	8	0	9
7	121	0	11	0	11
8	118	0	13	0	13
9	117	0	19	0	19
10	116	2	23	0	25

**Table 5:** Pareto optimal frontier solutions generated when *k_f_* = 20.

No.	*nm*_1_	*nm*_2_	*nm*_3_	*nm*_4_	*nm*_5_
1	129	0	2	0	2
2	128	0	4	0	4
3	127	2	4	0	6
4	124	3	4	0	7
5	120	0	8	0	8
6	117	0	12	0	12
7	116	3	13	0	16

**Table 6:** Different $\Delta t_{\max}$ representative Pareto optimal frontiers solutions.

$\Delta t_{\max}$	No.	*nm*_1_	*nm*_2_	*nm*_3_	*nm*_4_	*nm*_5_
2	1	58	4	27	0	31
4	1	86	1	20	0	21
4	2	74	4	18	0	22
6	1	100	1	10	0	11
6	2	89	2	11	0	13
8	1	118	3	3	0	6
8	2	108	3	5	0	8
8	3	104	1	9	0	10
10	1	155	0	4	0	4
10	2	140	1	4	0	5
10	3	133	0	6	0	6
10	4	129	1	6	0	7
12	1	165	0	3	0	3
12	2	154	0	4	0	4
12	3	140	0	5	0	5

**Table 7:** The different Pareto optimal frontiers of $\Delta t_{\max}$ with the inclusion of the repair operator.

$\Delta t_{\max}$	No.	*nm*_1_	*nm*_2_	*nm*_3_	*nm*_4_	*nm*_5_
6	1	117	1	5	0	6
6	2	116	0	7	0	7
8	1	136	0	2	0	2
8	2	134	2	2	0	4
10	1	152	0	3	0	3
10	2	151	1	3	0	4
10	3	147	1	4	0	5
12	1	196	0	2	0	2
12	2	182	1	2	0	3
12	3	166	0	4	0	4

**Table 8:** Optimal Solution Selected After Introducing the Repair Operator.

Vehicle Number	Scheme for Vehicle Entry into the Tunnel		Scheme for Vehicle Exit from the Tunnel	
	Departure Time	Operation Path	Departure Time	Operation Path
1	0	11-12-0-1-2-3-4-5-6-7	10	7-6-9-8-10-15-14-13-12-11
2	4	11-12-18-19-20-21-22-23-16-9-6-7	16	7-6-5-8-10-22-29-28-27-26-19-18-12-11
3	9	11-12-13-14-20-21-28-29-30-24-16-9-6-7	23	7-6-5-4-3-2-1-0-12-11
4	14	11-12-18-19-26-27-28-21-22-23-16-9-6-7	28	7-6-5-4-10-15-14-13-12-11
5	19	11-12-0-1-2-14-15-10-8-9-6-7	31	7-6-5-8-9-16-24-30-29-22-21-20-19-18-12-11
6	26	11-12-13-14-15-10-8-9-6-7	36	7-6-5-4-3-2-14-13-12-11
7	31	11-12-18-19-20-21-22-23-16-17	41	17-16-24-30-29-22-10-15-14-13-12-11
8	38	11-12-13-14-15-10-22-23-16-17	48	17-16-24-30-29-28-21-20-14-13-12-11
9	46	11-12-13-14-15-10-8-9-16-17	56	17-16-23-22-10-15-14-13-12-11
10	52	11-12-13-14-15-10-22-29-30-24-16-17	64	17-16-9-8-10-15-14-13-12-11
11	58	11-12-13-14-15-10-22-23-16-17	68	17-16-24-30-29-28-27-26-19-18-12-11
12	62	11-12-13-14-15-10-8-9-16-17	72	17-16-23-22-10-15-14-13-12-11
13	66	11-12-13-14-15-10-22-29-30-24-25	77	25-24-16-9-6-5-4-10-15-14-13-12-11
14	74	11-12-13-14-15-10-22-23-30-24-25	85	25-24-16-23-30-29-28-21-20-14-13-12-11
15	82	11-12-13-14-15-10-8-9-16-24-25	93	25-24-30-23-22-21-20-14-13-12-11
16	86	11-12-13-14-15-10-22-29-30-23-16-24-25	99	25-24-30-29-28-27-26-19-18-12-11
17	94	11-12-13-14-15-10-4-5-6-9-16-24-25	107	25-24-30-23-22-21-20-14-13-12-11
18	98	11-12-13-14-15-10-8-9-16-24-25	109	25-24-30-29-28-27-26-19-18-12-11
19	106	11-12-13-14-20-21-22-23-16-24-25	117	25-24-30-23-16-9-6-5-4-10-15-14-13-12-11
20	114	11-12-13-14-20-21-22-23-16-24-25	125	25-24-30-29-28-27-26-19-18-12-11

## Data Availability

The original contributions presented in this study are included in the article. Further inquiries can be directed to the corresponding authors.
